# Supplementary material for: Small molecule activation of metabolic enzyme pyruvate kinase muscle isozyme 2, PKM2, circumvents photoreceptor apoptosis
Source: Sci Rep. 2020 Feb 19;10:2990. doi: 10.1038/s41598-020-59999-w (PMC7031539; doi:10.1038/s41598-020-59999-w)
Supplement: Supplementary file 1 — Supplementary information. [file 41598_2020_59999_MOESM1_ESM.pdf]

Small molecule activation of metabolic enzyme pyruvate kinase muscle isozyme 2, PKM2, circumvents photoreceptor apoptosis

Thomas J. Wubben, MD, PhD[1]; Mercy Pawar, MS[1]; Eric Weh, PhD[1]; Andrew Smith, BS[1]; Peter Sajjakulnukit, BS[2]; Li Zhang, PhD[3]; Lipeng Dai, PhD[4]; Heather Hager, MS[1]; Manjunath P. Pai, PharmD[4]; Costas A. Lyssiotis, PhD[2]; *Cagri G. Besirli, MD, PhD[1]

Affiliations: [1]University of Michigan, Kellogg Eye Center, Department of Ophthalmology and Visual Sciences; [2]University of Michigan, Department of Molecular and Integrative Physiology; [3]University of Michigan Biomedical Research Core Facilities, Metabolomics Core; [4] University of Michigan, College of Pharmacy

Address correspondence to: Cagri G. Besirli; University of Michigan, Kellogg Eye Center 1000 Wall St, Ann Arbor, MI 48105, cbesirli@med.umich.edu, phone: (734)-232-8404

Figure S1. Metabolic comparison between wild-type mouse and rat retina. (a) Volcano plot showing significantly altered metabolites between wild-type C57BL/6J mouse retina and Brown-Norway rat retina. The vertical dashed lines represent a fold change of 1.5 and the horizontal dashed line depicts a p-value of 0.05. (b) Metabolic pathway impact analysis of significantly altered metabolites by Metaboanalyst 4.0 in wild-type C57BL/6J mouse retina as compared to wild-type Brown-Norway rat retina.

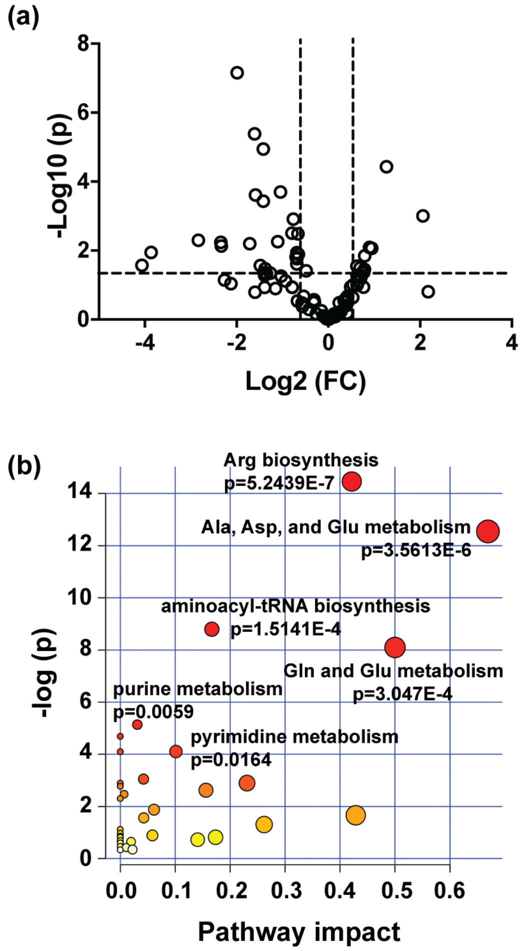
(a)
-Log10 (p)
Log2 (FC)
(b)
Arg biosynthesis
p=5.2439E-7
Ala, Asp, and Glu metabolism
p=3.5613E-6
aminoacyl-tRNA biosynthesis
p=1.5141E-4
Gln and Glu metabolism
p=3.047E-4
purine metabolism
p=0.0059
pyrimidine metabolism
p=0.0164
-log (p)
Pathway impact

Table S1. List of metabolites and their parameters detected by LC/MS.

Compound Name	Precursor Ion (Da)	Product Ion (Quantifier) (Da)	Product Ion (Qualifier) (Da)	Retention Time (min)	Collision Energy (Volts)
±-Mevalonolactone	189.1	59.1		2.91	6
2-2-Dimethyl Succinic acid	145.1	101.1		14	11
	145.1		127.1	14	8
2-3-Dihydroxybenzoic acid	153	109		14.5	15
	153		53.2	14.5	25
2-3-Dihydroxyisovalerate	133.1	75.1		10.8	10
	133.1		57.2	10.8	15
2-3-Pyridinedicarboxylic acid	166	122		14.9	6
	166		78.1	14.9	13
2-4-Quinolinediol	160	117.9		15.2	19
	160		42.2	15.2	29
2-Deoxyadenosine	250	134.05		6.5	12
	310.1	250		6.5	4
2-Deoxyadenosine 5-diphosphate	410	79.1		15.7	44
	410		158.7	15.7	24
2-Deoxyadenosine 5-monophosphate	330	79		12.7	48
	330		134.05	12.7	28
2-Deoxycytidine	286.1	226		2	4
	286.1		93	2	20
2-Deoxycytidine 5-diphosphate	386	79		14.7	36
	386		97	14.7	20
2-Deoxycytidine 5-monophosphate	306	79		10.4	44
	306		194.9	10.4	14
2-Deoxy-D-glucose 6-phosphate	243	79.1		9.1	45
	243		97	9.1	14
2-Deoxy-D-ribose	193.1	59.2		1.4	16
	193.1		133	1.4	0
2-Deoxyguanosine	266.1	150		4	12
	266.1		108	4	36
2-Deoxyguanosine 5-diphosphate	426	79.1		15.4	48
	426		158.9	15.4	28
2-Deoxyguanosine 5-monophosphate	346	79.1		12.6	48
	346		97	12.6	36
2-Deoxyinosine	251.1	135		3.7	20
	251.1		108	3.7	42
2-Deoxyribose 5-phosphate	213	79.1		9.23	40
	213		96.9	9.23	12
2-Deoxyuridine	227.1	184		2.7	6
	227.1		42.2	2.7	20
2-Deoxyuridine 5-triphosphate	467	158.8		16.8	36
	467		368.9	16.8	20
2-Isopropylmalic acid	175.1	113		15.5	13
	175.1		115	15.5	13
2-Ketobutyrate	101	57.2		12.4	4
2-Methyl-1-butanol	87.1	43.3		9.6	5
2-Phosphoglyceric acid	185	97		14.8	14
	185		79	14.8	37
3-2-Hydroxyethylindole	160.1	130.06		15.4	14
	160.1		142.07	15.4	16
3-Dehydroshikimic acid	171	127		7.4	8
	171		109	7.4	17

3-Hydroxyanthranilic acid	152	108		12.1	12
	152		107	12.1	23
3-Hydroxy-DL-kynurenine	223.1	205.9		2.3	4
	223.1		162	2.3	10
3-Hydroxyphenylacetic acid	151	107.1		14.2	4
	151		65.2	14.2	26
3-Indoleacetic acid	174.1	130		16.5	7
	174.1		128	16.5	19
3-Methylglutaric acid	145.1	101.1		14.1	11
	145.1		41.3	14.1	52
4-Aminobenzoic acid	136	92		9.8	8
4-Guanidobutyric acid	144.1	102		1.4	8
	144.1		41.2	1.4	32
4-Hydroxybenzoic acid	137	93		12.1	14
	137		65.2	12.1	34
4-Hydroxy-L-glutamic acid	162	144		5.3	6
	162		72.1	5.3	16
4-Hydroxyphenyl-pyruvic acid	179	107		14.7	4
4-Methyl-2-oxovaleric acid	129.1	85.1		13.7	7
4-Pyridoxic acid	182	138		15.3	12
	182		108.1	15.3	20
4-Quinolinol	144	66.1		9.2	44
	144		116	9.2	28
5-Deoxy-5-(methylthio)adenosine	356.1	134		11.8	12
	356.1		296	11.8	4
5-Hydroxy-3-indoleacetic acid	190.1	144		13	21
	190.1		116	13	46
5-Methoxytryptamine	189.1	144		2.4	28
	189.1		174	2.4	12
6-Hydroxynicotinic acid	138	94.1		9	10
	138		42.3	9	27
Adenine	134	107		2.8	18
	134		92.1	2.8	20
Adenosine	266	134		6.4	10
	326.1	134		6.4	20
Adenosine 3-5-cyclic monophosphate	328	134		13.4	24
	328		79	13.4	48
Adenosine 5-diphosphate	426	159		15.4	28
	426		328	15.4	16
Adenosine 5-monophosphate	346	79		11.6	38
	346		97	11.6	24
Adenosine 5-triphosphate	506	408.1		17.2	22
	506		159	17.2	38
Adenylosuccinic acid	462	97		16.4	24
	462		134.05	16.4	48
Adipic acid	145.1	101.1		13.9	11
	145.1		83.1	13.9	12
AICAR	257.1	125		2.3	10
	257.1		42.2	2.3	44
Allantoin	157	42.2		1.3	16
	157		97	1.3	12
alpha-D(+)Mannose 1-phosphate	259	79		7.6	48
	259		97	7.6	14
alpha-D-Glucose-1-phosphate	259	79		7.6	28
	259		240.9	7.6	9
alpha-Ketoglutaric acid	145	101		14.3	5

	145		57.2	14.3	9
Arabinose-5-phosphate	229	79		8.4	36
	229		97	8.4	8
Argininosuccinic acid	289	88		4.6	36
	289		131.1	4.6	27
beta-Nicotinamide adenine dinucleotide	662.1	540		9.5	12
	662.1		327.9	9.5	36
beta-Nicotinamide mononucleotide	333	251.1		3.7	12
	333		135.1	3.7	36
Cellobiose	341.1	161		1.3	5
Chorismic acid	225	189		6.7	6
cis-Aconitic acid	173	129		15.5	4
	173		85.1	15.5	8
Citramalic acid	147	85.1		13.8	12
	147		87	13.8	14
Citric acid	191	111.01		14.9	10
	191		87.01	14.9	16
CoA	766.1	408.1		17.8	40
	766.1		686.2	17.8	40
Creatine	130.1	88.1		1.3	8
	130.1		41.2	1.3	36
Creatine phosphate	210	79		7	16
	210		96.9	7	4
Creatinine	112	41.2		1.35	24
	112		68.1	1.35	16
Cysteine	120	33.2		1.31	10
Cytidine	242.1	109		1.8	8
	242.1		42.2	1.8	16
Cytidine 5-diphosphate	402.01	79		14.4	48
	402.01		158.92	14.4	24
Cytidine 5-triphosphate	482	158.8		17	40
	482		79	17	40
Cytidine-5-monophosphate	322	79		9.7	44
	322		97	9.7	22
Cytosine	110	67.1		1.4	8
	110		42.2	1.4	12
D-+-Galactosamine	238.1	159.9		1.1	8
	238.1		100	1.1	6
Deoxyadenosine 5-triphosphate	490	391.9		17.2	24
	490		158.9	17.2	32
Deoxycytidine 5-triphosphate	466	158.9		16.8	28
	466		367.9	16.8	20
Deoxyguanosine 5-triphosphate	506	407.9		17.2	20
	506		158.8	17.2	32
Deoxythymidine 5-triphosphate	481	158.8		17.2	36
	481		383.1	17.2	20
D-erythro-Dihydrosphingosine	300.3	199		8.9	8
	300.3		282	8.9	6
D-Fructose 1,6-biphosphate	338.9	97		14.8	22
	338.9		241.01	14.8	12
D-Fructose 6-phosphate	259	79		7.6	48
	259		97	7.6	14
D-Gluconic acid	195.1	75.2		5.9	18
	195.1		129	5.9	10
D-Glucosamine 6-phosphate	258	79		1.6	48
	258		97	1.6	16

D-Glucose 6-phosphate	259	79		7.6	48
	259		97	7.6	14
Dihydroxyacetone phosphate	169	79		10.5	32
	169		97	10.5	6
DL-2-Aminoadipic acid	160.1	142		4.3	9
	160.1		116	4.3	12
DL-Glyceraldehyde 3-phosphate	169	79		11.7	28
	169		97	11.7	4
DL-Isocitric acid	191	111.02		15.1	11
	191		173	15.1	6
D-Maltose	341.1	161		1.3	5
	341.1		179	1.3	4
D-Mannose	179.1	89		1.3	4
	179.1		59.2	1.3	16
D-pantothenic acid	218	88		12.1	10
	218		146.08	12.1	14
D-Ribose 5-phosphate	229	79		8.5	48
	229		97	8.5	10
D-Ribulose 1,5-biphosphate	308.9	79		14.7	46
	308.9		97	14.7	22
D-Sedoheptulose-7-phosphate	289	96.9		8.2	18
	289		79	8.2	48
D-Xylose	149.05	89.02		1.2	4
	149.05		71.01	1.2	4
D-Xylulose-5-phosphate	229	79		8.5	36
	229		138.98	8.5	8
Epicatechin	289.1	245		12.2	11
	289.1		109	12.2	26
Flavin adenine dinucleotide	784.1	346		17	35
	784.1		437	17	30
Folinic acid	472.2	315		14.8	28
	472.2		343	14.8	20
Galactonic acid	195.1	75.1		5.9	18
	195.1		129	5.9	11
gamma-Aminobutyric acid	102	84.1		1.2	8
gamma-Glu-Cys	249.1	128		8.1	8
	249.1		171	8.1	9
Glucoheptonic acid	225.1	129		5.8	11
	225.1		75.1	5.8	18
Glutathione Reduced	306.1	143		8.43	17
	306.1		128.1	8.43	17
Glyceric acid	105	75.1		5.9	8
	105		57.2	5.9	14
Glyoxylic acid	73	45.2		5.9	6
Guanine	150	133.02		2	12
	150		66.01	2	36
Guanosine	282.1	150		3.9	17
	282.1		132.9	3.9	32
Guanosine 3,5-cyclic monophosphate	344	150.042		12.2	24
	344		133.02	12.2	44
Guanosine 5-diphosphate	442	150		14.8	31
	442		158.9	14.8	20
Guanosine 5-triphosphate	522.3	158.9		17	36
	522.3		424	17	20
Homocitrate	205	125		15.4	10
	205		145	15.4	10

Hypoxanthine	135	92		2	17
	135		65.1	2	28
Indoline-2-carboxylate	162.1	116		16.1	16
	162.1		118	16.1	11
Inosine	267.1	135		3.3	22
	267.1		108	3.3	44
Inosine 5-diphosphate	427	158.9		14.5	28
	427		135	14.5	24
Inosine 5-monophosphate	347	79		11.1	44
	347		97	11.1	22
Inosine 5-triphosphate	507	409		17.2	19
	507		159	17.2	40
Isopentenyl pyrophosphate	245	209		12.8	4
Isopentyl acetate	129.1	85.2		14.6	6
	129.1		41.2	14.6	12
Itaconic acid	129	85.1		13.6	6
	129		41.3	13.6	12
Ketoisovaleric acid	115	71.2		16.56	4
Ketovaleric acid	115	71.2		14.5	4
Lactic acid	89	43.3		7.4	10
	89		45.3	7.4	9
L-Arabinose	149	89.1		1.4	4
	149		59.2	1.4	12
L-Arabitol	151	89		1.3	12
	151		71.02	1.3	16
L-Arginine	173.1	131		1.1	11
	173.1		156	1.1	8
L-asparagine	131	113		1.2	4
	131		70	1.2	12
L-Aspartic Acid	132	88		4.9	10
	132		71	4.9	14
L-Canavanine	175.1	74.04		1.1	10
	175.1		118	1.1	8
L-Carnitine	220.1	146		1.2	4
L-Citrulline	174.1	131		1.3	8
	174.1		42.2	1.3	48
L-Cystathionine	221.1	134		1.3	8
	221.1		120	1.3	9
L-Cystine	239	74.1		1.2	20
	239		120	1.2	4
L-Dihydroorotic acid	157	113		7.3	5
	157		42.2	7.3	30
L-Glutamic acid	146	102		4.5	11
	146		128	4.5	8
L-Glutamine	145.1	127		1.3	7
	145.1		109	1.3	10
L-Glutathione (oxidized)	611.1	306.07		13.6	24
	611.1		272.09	13.6	28
L-Histidine	154.1	137		1.1	12
	154.1		93.1	1.1	16
L-Homocysteine	134	116.9		1.4	8
L-Homocystine	267	132		1.5	8
	267		115	1.5	16
L-Homoserine	118	72.1		1.3	10
	118		55	1.3	16
L-Hydroxyglutaric acid	147	128.9		13.8	7

	147		85.1	13.8	13
Lipoamide	204.1	158		16.8	4
	204.1		64.1	16.8	20
L-Isoleucine	130	45		2.1	16
L-Kynurenine	207.1	190		3.9	4
	207.1		144	3.9	20
L-Leucine	130	45		2.1	16
L-Malic acid	133	115		13.8	8
	133		71.1	13.8	14
L-Methionine	148	47.2		1.8	12
	148		100	1.8	6
L-Phenylalanine	164.1	147		4.5	9
	164.1		103	4.5	15
L-Proline	114	68.1		1.3	12
	114		45.2	1.3	12
L-Serine	104	74.1		1.2	8
L-Sorbose	179.1	89		1.3	4
	179.1		71.02	1.3	14
L-Threonine	118	74.1		1.3	9
L-Tryptophan	203.1	116		7.9	14
	203.1		74.1	7.9	14
L-Tyrosine	180	119		2.3	15
	180		93	2.3	12
Maleic acid	115	71.2		12.8	7
	115		27.3	12.8	15
Malonic acid	103	59.2		12.8	7
	103		41.3	12.8	32
Melibiose	341.1	179		1.3	4
	341.1		89	1.3	13
Mevalonic acid	147.1	128.9		9.8	8
	147.1		103.1	9.8	11
Mevalonic acid 5-phosphate	227	79		14.8	40
	227		97	14.8	12
m-Hydroxybenzoic acid	137	93.1		13.4	10
	137		65.2	13.4	28
myo-Inositol	179	71.02		1.3	12
	239.1	179		1.3	0
N-Acetyl D-galactosamine	220.1	119		1.2	0
N-Acetyl-alpha-D-glucosamine 1-phospha	300	97		8.7	16
	300		79	8.7	36
N-acetylaspartate	174	88.1		14	14
	174		58.1	14	22
N-acetylaspartylglutamate	303.1	285		16.1	6
	303.1		128	16.1	14
N-Acetyl-D-glucosamine 6-phosphate	300	97		8.8	18
	300		79	8.8	48
N-Acetylglutamic acid	188.1	128.04		14.1	10
	188.1		102	14.1	16
N-Acetylneuraminic acid	308.1	87		6.2	14
	308.1		169.9	6.2	10
N-Carbamoyl-DL-aspartic acid	175	132		13.5	7
	175		88.1	13.5	19
N-Carbamyl-L-glutamic acid	189.1	146		13.6	7
	189.1		102.1	13.6	20
N-Formyl-L-Tyrosine	208.1	164		12.8	7
	208.1		107	12.8	12

Nicotinic acid	122	78.1		11.9	11
	122		51.2	11.9	32
Nicotinic acid mononucleotide	334	289.9		8.6	4
	334		79	8.6	40
o-Hydroxy hippuric acid	194	150		16.5	11
	194		121	16.5	22
o-Phospho-L-Serine	184	79		10.2	36
	184		97	10.2	13
O-Phosphorylethanolamine	140	79		1.7	14
	140		63	1.7	38
Orotic acid	155	42		9.1	26
	155		40	9.1	44
O-Succinyl-L-homoserine	218.1	117		6.5	7
	218.1		118	6.5	0
Oxamic acid	88	44.3		6.5	6
	88		42.3	6.5	12
Phenylpyruvic acid	163	91.1		17.2	6
Phosphoenolpyruvic acid	167	79		15.3	12
Prephenic acid	225	101		12.4	9
Pyridoxal 5 phosphate	246	79		14.8	30
Pyridoxal hydrochloride	166.1	138		2.4	12
	166.1		108	2.4	18
Pyridoxamine	167.1	121		1.1	22
	167.1		122	1.1	18
Pyridoxine	168.1	122.1		2.4	17
	168.1		166	2.4	9
Pyruvic acid	87	43.2		9.5	4
Quinic acid	191.1	85.1		6	25
	191.1		93.1	6	24
Riboflavin	375.1	255		12.6	14
	375.1		212	12.6	28
Ribonic acid gamma lactone	147	59.2		1.5	16
	147		99	1.5	8
S-2-Aminoethyl-L-cysteine	163.1	76.1		1.1	10
	163.1		33.2	1.1	28
S-5-Adenosyl-L-homocysteine	383.1	134		5.3	24
	383.1		248	5.3	13
Salicylic acid	137	93.1		16.8	17
	137		65.2	16.8	33
Shikimic acid	173	111		5.8	7
	173		93.1	5.8	16
Succinic acid	117	73.1		13	10
	117		99	13	8
Succinic semialdehyde	101	57.2		7.1	7
Taurine	124	80		1.3	24
Taurocholic acid	514	514		20.7	50
Thiamine	264.1	234		1.1	6
	264.1		148	1.1	18
Thymidine	241.1	42.2		5.5	16
	241.1		151	5.5	6
Thymidine 5-diphosphate	401.01	97		15.4	25
	401.01		79	15.4	44
Thymine	125	42.2		2.7	14
trans-4-Hydroxy-L-proline	130.1	128		1.3	12
	130.1		66.03	1.3	16
trans-Aconitic acid	173	129		15.55	4

	173		85.1	15.55	10
trans-trans Muconic acid	141	53.3		14.7	8
Trehalose	341	89.01		1.3	12
	401.1	341		1.3	10
Trehalose 6-phosphate	421.1	241		7.9	27
Uracil	111	42.2		1.7	14
Uric acid	167	124		5.5	13
	167		42.3	5.5	26
Uridine	243.1	200		2.3	6
	243.1		110	2.3	12
Uridine 5-diphosphate	403	158.9		14.8	28
	403		79	14.8	48
Uridine 5'-diphosphogalactose	565	322.9		14.3	24
	565		384.9	14.3	29
Uridine 5-diphosphoglucose	565	322.9		14.3	24
	565		384.9	14.3	28
Uridine 5-monophosphate	323	79.1		10.8	48
	323		97	10.8	24
Uridine 5-triphosphate	483	158.9		17	36
	483		385	17	20
Vanillic acid	167	123		12.6	9
	167		108	12.6	18
Xanthine	151	108		2.1	16
	151		42.2	2.1	24
Xanthosine	283.1	151		8.2	18
	283.1		108	8.2	40
Xylitol	151.1	89		1.3	8
	151.1		71	1.3	8
